# Genetic Polymorphisms on TNFA, TNFRSF1A, and TNFRSF1B Genes Predict the Effectiveness of Anti-TNF-α Treatment in Inflammatory Bowel Disease Patients

**DOI:** 10.3390/biomedicines13030669

**Published:** 2025-03-08

**Authors:** Michelangelo Rottura, Igor Pirrotta, Domenico Antonio Giorgi, Natasha Irrera, Vincenzo Arcoraci, Federica Mannino, Rosario Campisi, Chiara Bivacqua, Laura Patanè, Giuseppe Costantino, Socrate Pallio, Walter Fries, Anna Viola, Giovanni Pallio

**Affiliations:** 1 Department of Clinical and Experimental Medicine, University of Messina, Via C. Valeria, 98125 Messina, Italy; mrottura@unime.it (M.R.); ipirrotta@unime.it (I.P.); domenicoantonio.giorgi@unime.it (D.A.G.); nirrera@unime.it (N.I.); varcoraci@unime.it (V.A.); chiarabiva@gmail.com (C.B.); laurapatane206@gmail.com (L.P.); spallio@unime.it (S.P.); walter.fries@unime.it (W.F.); 2 Department of Medicine and Surgery, University of Enna “Kore”, Contrada Santa Panasia, 94100 Enna, Italy; federica.mannino@unikore.it; 3 Department of Biomedical and Dental Sciences and Morphological and Functional Imaging, University of Messina, Via C. Valeria, 98125 Messina, Italy; rosario.c@lccampisigroup.it (R.C.); gpallio@unime.it (G.P.); 4 Department of Health Promotion, Mother & Child Care, Internal Medicine & Medical Specialties, Gastroenterology & Hepatology Unit, University of Palermo, Piazza delle Cliniche, 90127 Palermo, Italy; 5 IBD-Unit, University Hospital Gaetano Martino, Via C. Valeria, 98125 Messina, Italy; giu.costantin@gmail.com

**Keywords:** inflammatory bowel diseases, Crohn’s disease, ulcerative colitis, infliximab, adalimumab, polymorphisms, precision medicine

## Abstract

**Background/Objectives:** Tumor necrosis factor alpha (TNF-α) is the key inflammatory cytokine involved in the pathogenesis of inflammatory bowel diseases (IBDs). Anti-TNF-α therapy has been successfully used for IBD treatment, although the therapeutic response differs among patients due to the genetic background. The aim of this study was to investigate whether the presence of single nucleotide polymorphisms (SNPs) on *TNFA*, *TNFRSF1A*, and *TNFRSF1B* genes could affect anti-TNF-α treatment effectiveness in IBD patients. **Methods:** In this prospective cohort study, 83 European IBD patients treated with infliximab or adalimumab (with or without steroid bridge therapy) as first-line therapy were enrolled. Genomic DNA was extracted from peripheral blood, and *TNF-α* (rs1800629, rs361525, rs1799724), *TNFRSF1A* (rs767455), and *TNFRSF1B* (rs1061622, rs1061624, rs3397, rs976881) SNPs were assessed. Steroid-free remission (SFR) (clinical remission together with steroid interruption) and anti-TNF-α therapy persistence after 12 months of follow-up were evaluated. Patients who stopped anti-TNF-α therapy before the end of follow-up, due to side effects or treatment failure, were defined as discontinuers. **Results:** A higher frequency of the G/G genotype in rs1800629 and the A/A genotype in rs1061624 was observed in the SFR group compared to non-SFR (97.7% vs. 82.8%; *p* = 0.025 and 32.6% vs. 10.3%; *p* = 0.029, respectively). Moreover, carriers of the A/A genotype in rs361525 and the C/C genotype in rs767455 had a lower probability of achieving SFR than wild-type patients (OR = 0.14; 95% CI= 0.03–0.69; *p* = 0.016 and OR = 0.10; 95% CI = 0.02–0.60; *p* = 0.012, respectively). Furthermore, an increased frequency of rs1800629 A allele was observed in patients who discontinued treatment compared to completers (27.3% vs. 6.9%; *p* = 0.033), as well as a high risk of interrupting therapy (HR = 6.47; 95% CI = 1.15–36.38). **Conclusions:** These results suggest that the evaluation of SNPs in *TNF-α*, *TNFR1A*, and *TNFR1B* genes could improve the management of IBD, leading to more effective, individualized treatment plans and a reduction in healthcare costs associated with ineffective therapies and disease complications.

## 1. Introduction

Inflammatory bowel diseases (IBDs), including Crohn’s disease (CD) and ulcerative colitis (UC), are chronic inflammatory conditions of the gastrointestinal tract, with 3.32 million estimated cases in 1990 and 4.90 million cases in 2019, corresponding to an increase of 47.45% between 1990 and 2019, with a prevalence of 84.3 (79.2–89.9) per 100,000 population [1,2,3,4]. Genetic and environmental factors may influence immune response and related diseases, including CD and UC [5].

In the last decade, the goal of pharmacological treatments has not been limited exclusively to symptom control but has also aimed at “deep remission”, which includes both clinical remission and mucosal healing, in order to prevent disease progression and disability. Anti-inflammatory drugs (aminosalicylates, mesalazine, sulfasalazine, and systemic corticosteroids), antibiotics (amoxicillin, ciprofloxacin, metronidazole, and azithromycin), and immunomodulators (azathioprine, mercaptopurine, and methotrexate) are the main drugs used for IBD treatment and may be prescribed alone or in combination as the mainstay of medical management [6]. Surgery may be necessary when drug therapy is not effective in controlling the disease or in the presence of complications, such as intestinal obstruction, fistulas, or severe bleeding. However, surgery is not curative, and post-operative endoscopic recurrence occurs in more than half of patients within 1 year, especially in CD [7]. In recent years, the use of biotechnological drugs for IBD treatment has increased significantly. The most commonly prescribed biological agents include anti-TNF-α antibodies (infliximab and adalimumab), anti-integrin (vedolizumab), IL-12 and IL-23 inhibitors (ustekinumab), and anti-IL-23 inhibitors (risankizumab and mirikizumab). These drugs have revolutionized the treatment of IBDs by offering effective therapeutic options for patients who do not respond to conventional treatments or who have severe disease activity. In particular, the most widely used biological drugs for the treatment of IBDs are infliximab (a chimeric murine IgG1 monoclonal antibody targeting TNF-α) and adalimumab (a fully humanized IgG1 monoclonal antibody against TNF-α) [8]. However, around 25% of patients experience treatment failure within 1 year of starting a first-line biologic for either UC or CD [9]. A further number of patients who initially respond to treatment eventually lose reactivity, a phenomenon called secondary non-response. Therefore, clinical response may vary from patient to patient, and early identification of treatment failure with anti-TNF-α agents is of significant importance from both a clinical and an economic point of view. Thus, robust data have demonstrated that early treatment of IBDs is critical to prevent complications and, in particular, real-world data have shown that biologic treatment within 2 years of diagnosis was associated with higher rates of remission and mucosal healing compared to late or non-biologic treatment [10].

Shorter disease duration, isolated colon disease, younger age, and non-smoking status represent the main factors associated with anti-TNF-α treatment success. Conversely, clinical failure may be due to an alternative non-TNF-α-mediated inflammatory pathway, the development of anti-drug antibodies, or the presence of genetic polymorphisms. Improvements in genetic characterization techniques and genome-wide association studies (GWAS) have allowed for the identification of genetic variants that could influence both disease development and response to treatment, as well as the occurrence of adverse events [11,12]. Several genes have been implicated in the pathogenesis of IBDs. However, limited evidence exists regarding the ability to predict response to anti-TNF-α treatment in CD and UC based on genetic data. Some studies have shown that single nucleotide polymorphisms (SNPs) in the genes encoding TNF-α or its receptors, TNFR1A and TNFR1B, can influence the response to anti-TNF-α drugs in patients with IBDs, although some results appear controversial [13,14,15,16]. Therefore, the aim of the present study was to investigate eight SNPs within the genes encoding TNF-α, TNFR1A, and TNFR1B to evaluate whether their presence could influence treatment effectiveness with infliximab or adalimumab in patients with CD and UC in order to allow for efficient, personalized, and cost-effective treatment.

## 2. Material and Methods

### 2.1. Patients Recruitment and Data Collection

A prospective cohort study was conducted by enrolling 83 patients from the IBD Unit of the A.O.U. “G. Martino” of Messina in the period between 1 May 2022 and 1 May 2024. Patients affected by UC and CD who started treatment with infliximab or adalimumab were selected and monitored for 12 months after the start of biological therapy. The clinicians involved in the study provided a complete and exhaustive explanation of the protocol at the time of enrollment and collected written informed consent. The study was approved by the Ethics Committee of Messina with the identification code n° 41/22 of 12 April 2022.

The following sociodemographic and clinical characteristics were recorded: gender, smoking, IBD family history, date of diagnosis and start date of biological therapy, type of biological therapy (infliximab or adalimumab), any cause of treatment interruption, concomitant therapies, and comorbidities. Patients were managed according to clinical practice every two months (with close time intervals during the induction of therapy, as per the drug label, or in the case of treatment optimization). At the 12-month follow-up, clinical remission together with the interruption of steroidal bridge therapy were evaluated.

Clinical activity was evaluated by using clinical scores, such as the Harvey Bradshaw Index (HBI) for CD and the partial Mayo Score (pMS) for UC. In particular, steroid-free remission (SFR) was defined as clinical disease activity with a HBI ≤ 5 points for CD and a pMS ≤ 2 without any kind of concomitant steroids.

Patients with SFR were included in the “SFR group”, and all others were included in the “non SFR group”. Patients who discontinued anti-TNF-α treatment due to side effects or treatment failure before the end of follow-up were defined as “discontinuers”.

### 2.2. SNPs Selection, DNA Extraction, and Genotyping

A PubMed literature search was conducted by using the keywords “tumor necrosis factoralpha”, “anti-TNF-α”, “infliximab”, “adalimumab”, “polymorphism”, “SNP”, “ulcerative colitis”, and “Crohn’s disease” using Boolean operators (AND), (OR), (NOT). Results were restricted to original studies that investigated SNPs and included allele frequencies and genotypes for different groups. SNPs that had biological relevance, those with an expected minor allele frequency ≥ 5%, and those that demonstrated an association with CD or UC and anti-TNF-α treatment were selected. SNPs were excluded if they had been extensively investigated and if there was no prognostic value for the combination of CD or UC and anti-TNF-α treatment response. As a result of this search, the following eight SNPs within 3 genes of *TNF-α* (rs1800629, rs361525, rs1799724), *TNFRSF1A* (rs767455), and *TNFRSF1B* (rs1061622, rs1061624, rs3397, rs976881) were analyzed. Peripheral blood was collected through venipuncture in 3 mL tubes containing EDTA as the anticoagulant. Genomic DNA was extracted from blood samples by using a standard protocol. In summary, cells were lysed in a DNA buffer containing 10 mM Tris-HCl (pH 7.4), 100 mM NaCl, 1 mM EDTA, and 1 mM MgCl_2_. Following centrifugation, the nuclei were lysed overnight at 37 °C with 10% SDS and proteinase K. Organic solvents, phenol, and chloroform were used to remove cell debris, primarily proteins. DNA was then washed with 70% ethanol and resuspended in an appropriate volume of RNase/DNase-free water. Its concentration was measured spectrophotometrically for each sample and stored at –20 °C until use for genotyping assay [17]. SNP assessment was performed by using TaqMan predesigned genotyping assays (Thermo Fisher Scientific, Waltham, MA, USA). The TaqMan genotyping assays were diluted to a 20× working stock solution with 1× TE buffer (10 mM Tris-HCl, 1 mM EDTA, pH 8.0, in DNase-free, sterile-filtered water), as recommended by the manufacturer. A MicroAmpTM Optical 96-Well Reaction Plate was used, and a total of 25 μL of reaction volume was used (12.50 μL of 2X TaqMan Master Mix, 1.25 μL of 20X assay working stock solution, 1 μL of DNA sample at a concentration of 10 ng/μL, 10.25 of nuclease-free water) for each well; the plate was then sealed and briefly centrifuged to bring the reaction mix to the bottom of the well and remove air bubbles. PCR reactions were performed with the QuantStudio6 Flex system (Applied Biosystems, Foster City, CA, USA), and the following program was used: 95 °C for 10 min, followed by 40 cycles of 95 °C for 15 s and then 60 °C for 1 min. The analysis was performed by using real-time instrument software (Applied Biosystems, Foster City, CA, USA), and each genotype was independently assigned by two investigators. In cases of disagreement, assignment was reached through consensus. Patient carriers of a homozygous major allele for each SNP were considered wild-type (WT).

### 2.3. Sample Size Calculation

A sample size calculation was performed by assuming an expected incidence of non-response equal to 25% after 12 months of anti-TNF-α treatment in IBD patients based on literature data [9] and an expected incidence of 40% for our study group, a probability error = 0.05 and a power level of 80%. Seventy-one patients represented the minimum sample size that guaranteed a powered study. However, 83 patients were enrolled considering possible patient drop-outs.

### 2.4. Statistical Analysis

A descriptive analysis of the demographic and clinical characteristics of the enrolled patients was performed. Characteristics of the “SFR group” were compared with the “non-SFR group”, and the characteristics of patients who discontinued treatment were compared with patients who completed the follow-up. The results were expressed as the median and the interquartile range (Q1–Q3) for continuous variables and as the absolute frequency and the percentage for categorical variables.

The variables studied did not have a normal distribution (as demonstrated by the Kolmogorov–Smirnov test); therefore, a non-parametric approach was used. Specifically, the Mann–Whitney test was used to compare continuous variables between independent groups, while the Pearson chi-square test was applied for categorical variables.

Univariate logistic regression models were used to identify predictive factors associated with clinical remission of the diseases. All variables identified as predictors were included in a stepwise multivariate logistic regression model (backward procedure, α = 5%), and odds ratios (OR) with their 95% confidence intervals (95% CI) were calculated.

Multivariate Cox regression models were used to assess the risk of biologic treatment discontinuation during the monitoring period, including covariate age, gender, biologic treatment (adalimumab or infliximab), diseases (UC or CD), steroid therapy at baseline, and genotypes. Results were reported as hazard ratios (HRs) with 95% CI.

Values of *p* < 0.05 were considered statistically significant, and all analyses were performed with SPSS version 23.0 software (IBM Corp., SPSS Statistics, Armonk, NY, USA).

## 3. Results

### 3.1. Patients Characteristics

A total of 83 patients were enrolled with a median age of 34 years (27–52 years), and 63.9% (N = 53) were male. In particular, 45 patients (54.4%) were affected by CD, with a median baseline HBI value of 3.0 (1.0–5.5), whereas 38 patients (45.8%) had UC, with a median PMS value of 2.5 (1.0–5.0). Furthermore, 51 patients (61.4%) were treated with infliximab, 32 patients (38.6%) were treated with adalimumab, and 49 (68.1%) also used steroid bridge therapy.

In total, 11 (13.3%) patients discontinued treatment with biologics early during the study period, and 72 patients (86.7%) continued the treatment for at least 12 months.

Among patients who completed the follow-up, 43 (59.7%) were in the SFR group, and 29 (40.3%) were in the non-SFR group. No significant difference was observed between the two groups considering demographic and clinical characteristics (Table 1).

All 83 patients included in the study were genotyped to assess whether the allele frequencies of SNPs *TNF-α* (rs1800629, rs361525, rs1799724), *TNFRSF1A* (rs767455), and *TNFRSF1B* (rs1061624, rs3397, rs976881) were consistent with those normally expected in a European population. Our results showed that the allele frequencies of rs361525 were not in agreement with the Hardy–Weinberg equilibrium, although the allele frequencies of all other SNPs were (Table 2).

### 3.2. SNPs Associated with Clinical Effectiveness

A higher frequency of the G/G genotype in rs1800629 was observed in the SFR group than in the non-SFR group (97.7% vs. 82.8%; *p* = 0.025). Furthermore, a higher frequency of the occurrence of the A/A genotype in rs1061624 was detected in the SFR group than in the non-SFR group (32.6% vs. 10.3%; *p* = 0.029 (Table 3).

The probability of achieving SFR was significantly reduced in patients with a homozygous mutated genotype in rs361525 (OR = 0.14; 95% CI = 0.03–0.69; *p* = 0.016) and in rs767455 (OR = 0.10; 95% CI = 0.02–0.60; *p* = 0.012) compared to WT patients for the same SNPs (Table 4).

### 3.3. SNPs Associated with Anti-TNF-α Treatment Discontinuation

No significant difference was observed between patients who discontinued treatment and patients who completed the follow-up considering the clinical and demographic characteristics. However, the heterozygous genotype in rs1800629 was more frequent in the group of patients who interrupted the treatment compared to patients who did not discontinue (27.3% vs. 6.9%; *p* = 0.033; Table 5).

**Figure 1:**
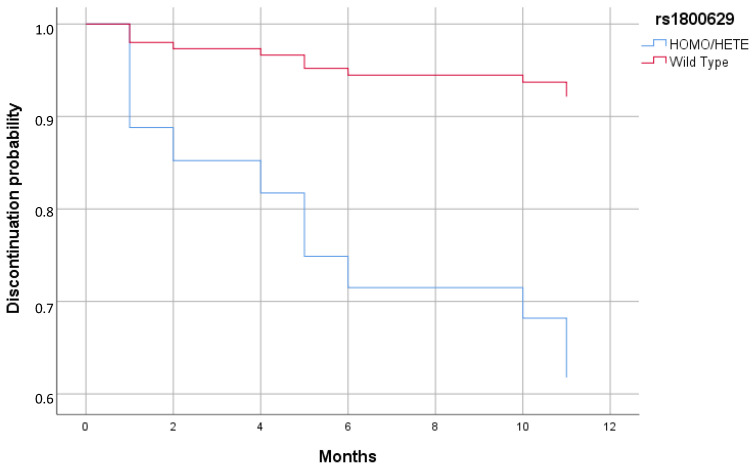
Cox regression of discontinuation of therapy stratified by rs1800629 minor allele (Homo/Hete) and WT.

Cox regression analysis reported a significant increased risk of discontinuing biologics in patient carriers of a minor allele in rs1800629 compared to patients with the WT genotype (HR = 5.98; 95% CI = 1.03–34.83; *p* = 0.047) (Figure 1 and Table 6).

## 4. Discussion

Nowadays, maximizing the therapeutic efficacy of anti-TNF-α biologic drug therapy and minimizing the risk of serious side effects by selecting patients who are likely to have a favorable outcome are of substantial clinical interest. Young age and concomitant immunosuppressive treatment have previously been associated with a short-term beneficial response to infliximab [18], while the development of anti-drug antibodies has been associated with a negative therapeutic response and an increased risk of severe infusion reactions [19,20]. Despite these findings, it has not yet been clarified whether the effectiveness and safety of biologic drugs in IBDs could be effectively predicted before the beginning of therapy. Genetic variation may characterize subgroups of patients with disparate efficacy and safety profiles due to distinct gene expression profiles [21].

To our knowledge, this is the first study that evaluates gene expression profiles in relation to a significant outcome, such as SFR. Previous studies have investigated only clinical response or clinical remission. In this study, eight SNPs in the *TNF-α*, *TNFRSF1A*, and *TNFRSF1B* genes were evaluated, and patients were stratified according to SFR achievement. Interestingly, carriers of the A/A genotype in rs1061624 appeared with a significantly higher frequency in the SFR group compared to the non-SFR group, and the G/G genotype in rs1800629 appeared with a significantly higher frequency in SFR than in the non-SFR group. The results regarding rs1061624 are not consistent with previous studies that demonstrated a correlation between the presence of a G variant in rs1061624 and a long-term response to infliximab [22], as well as an association between the A/T haplotype in rs1061624 and rs3397 and non-response to treatment in 297 Spanish CD patients undergoing infliximab treatment [23]. On the other hand, in accordance with the results of the present study, a previous paper has shown that the presence of a mutated genotype in rs1800629 was correlated with a three-fold increased risk of being a non-responder in a study conducted on 121 Caucasian patients with CD undergoing anti-TNF-α treatment [24]. An increased frequency of the rs1800629 A allele was also observed in non-responders to TNF-α inhibitors compared to responders in a study on 82 Spanish IBD patients [25], thus confirming the results obtained in our study showing that the WT genotype in this SNP appeared with a significantly higher frequency in the SFR group. On the other hand, several studies did not find an association between the presence of rs1800629 and clinical response either in adults or children [26,27,28,29,30], confirming that further validation is needed.

Furthermore, it has been demonstrated that the minor allele of the *TNFRSF1A* gene in rs767455 is associated with CD in Caucasians [31]. In our study, we aimed at investigating this polymorphism in relation to the effectiveness of anti-TNF-α biologic treatment in IBD patients evaluated with a strong outcome, such as SFR, thus observing that the presence of a homozygous mutated genotype is a predictive factor for non-achievement of SFR. This result was in accordance with previous findings obtained by Matsukura and colleagues in a Japanese population [32] and with the data reported by Pierik et al. that demonstrated an association with non-response evaluated based on serum C Reactive Protein (CRP) levels [33]. Instead, a study conducted on 444 patients with CD found no association between the presence of rs767455 and the clinical response to infliximab [34], just as no association was found in a study on 297 Spanish CD patients who underwent infliximab treatment [23].

SNPs in the gene encoding TNF-α have previously been associated with clinical response to anti-TNF-α biologic drugs. Specifically, a polymorphism at position −238 in the promoter region (rs361525) has previously shown conflicting data regarding the response to anti-TNF-α treatment in rheumatoid arthritis, psoriasis, and IBDs [25,35,36]. In our study, a homozygous genotype in rs361525 was correlated with a greater likelihood of being in the non-SFR group after infliximab or adalimumab treatment for 12 months. These results agree with a previous paper that linked this mutation to poor response to anti-TNF-α treatment in Danish patients with IBDs [37]. On the other hand, other previous studies did not find an association between the presence of rs361525 and clinical or biological response to infliximab treatment in CD or UC patients [25,27,30,38].

rs1799724 was also studied in our cohort, and no association was detected with the clinical outcomes, in accordance with the studies conducted by Duricova et al. and Papamichael et al. in adults and children affected by CD [28]. On the contrary, Matsuoka and colleagues demonstrated that the presence of this SNP could be considered a predicting factor for non-remission during infliximab maintenance therapy, at least in Japanese patients [30].

Moreover, in the present study, no correlation was observed with the presence of rs1061622, rs3397, or rs976881 in the *TNFRSF1B* gene with respect to anti-TNF-α treatment effectiveness, as previously demonstrated in other studies involving CD patients [30,32]. On the other hand, both Medrano et al. and Steenholdt et al. found an association between an increased frequency of the rs1061622 G carrier and disease remission in Caucasian CD patients [20,23], whereas Salvador-Martín et al. showed that rs3397 C allele presence was predictive of a longer time to failure in anti-TNF-α therapy [39]. Additionally, the research group of Steenholdt indicated that the minor allele carriage of rs976881 is associated with a loss of response to infliximab therapy [20].

We also investigated the possible association between the presence of SNPs and discontinuation of anti-TNF-α treatment. A significantly higher risk of treatment discontinuation was observed among heterozygosis carriers of the rs1800629 minor allele, but no result was reported in this study for homozygosis due to the low number of patients enrolled (only one patient in homozygosis). This result is partially at odds with the only other study in the literature regarding the effect of this mutation on treatment discontinuation conducted on psoriasis patients, which showed no significant difference in treatment discontinuation between WT and mutated patients [40].

The identification of SNPs in *TNF-α*, *TNFR1A*, and *TNFR1B* genes as potential predictors of response to infliximab or adalimumab in CD and UC has several important clinical implications. These findings could enhance treatment decision making in real-world settings by contributing to a more personalized approach to anti-TNF-α therapy by optimizing drug selection and treatment initiation. Thus, physicians integrating SNP profiling into routine clinical practice could predict whether a patient is likely to respond to infliximab or adalimumab before initiating treatment. This would allow for the selection of the most effective biologic therapy from the outset, reducing errors in prescribing and minimizing delays in achieving disease control. Moreover, unnecessary exposure to ineffective therapies could be avoided; thus, patients with genetic markers associated with poor response to anti-TNF-α agents could be redirected toward alternative biologic therapies, such as anti-integrins (vedolizumab), anti-interleukins (ustekinumab, risankizumab), or JAK inhibitors (tofacitinib, filgotinib, upadacitinib), earlier in their treatment journey. This would help in preventing prolonged disease activity, reducing adverse effects from ineffective medications, and decreasing the risk of complications. Furthermore, considering that biologic therapies are expensive and non-responders may require multiple treatment switches, leading to increased healthcare costs, the use of genetic tests for relevant SNPs could provide a cost-effective strategy by ensuring that only those likely to benefit receive anti-TNF-α agents, thus optimizing resource utilization.

Nevertheless, the present study presents some limitations. For instance, the relatively small cohort size and the limited ethnic diversity (only Caucasian patients) restrict the generalizability of results. Moreover, we did not evaluate potential confounders (e.g., concomitant medications) that could influence treatment response, and endoscopy was not performed to assess endoscopic activity or mucosal healing at the end of follow-up.

Ultimately, the evaluation of SNPs in *TNF-α*, *TNFR1A*, and *TNFR1B* genes could improve the management of IBD by enabling a precision medicine approach. This would lead to more effective, individualized treatment plans, improved patient outcomes, and a reduction in healthcare costs associated with ineffective therapies and disease complications. However, further validation in larger, multi-center cohorts with diverse patient populations is necessary before widespread clinical implementation.

## Figures and Tables

**Table 1:** Patients characteristics stratified by response to treatment (SFR vs. non-SFR).

Variable	Total	SFR	Non-SFR	*p*-Value
Patients	72	43	29	
Age (IQR); years	38 (28–51)	37 (26–52)	38 (29–52)	0.913
Gender (M)	47 (65.3%)	29 (67.4%)	18 (62.1%)	0.639
Diseases				
CD	40 (55.6%)	21 (48.8%)	19 (65.5%)	0.162
UC	32 (44.4%)	22 (51.2%)	10 (34.5%)	
Type of anti-TNF				
Infliximab	43 (59.7%)	29 (67.4%)	14 (48.3%)	0.104
Adalimumab	29 (40.3%)	14 (32.6%)	15 (51.7%)	
Baseline clinical activity				
HBI/PMS (IQR)	3 (1–5)	2 (1–5)	3 (1–5)	0.772
Steroid therapy	49 (68.1%)	27 (62.8%)	22 (75.9%)	0.243

**Table 2:** Genotype frequency and allele frequency in enrolled patients. 1: Major allele. 2: Minor allele (both in European populations). HWE: Hardy–Weinberg equilibrium.

Gene	rs Number	Genotype Frequency			Allele Frequency		HWE
					1	2	
*TNF-α*	rs1800629	G/G	G/A	A/A	G	A	
		74 (89.2%)	8 (9.6%)	1 (1.2%)	156 (94.0%)	10 (6.0%)	0.13
*TNF-α*	rs361525	G/G	G/A	A/A	G	A	
		48 (57.8%)	22 (26.5%)	13 (15.7%)	118 (71.1%)	48 (28.9%)	<0.01
*TNF-α*	rs1799724	C/C	C/T	T/T	C	T	
		64 (77.1%)	14 (16.9%)	5 (6.0%)	142 (85.5%)	24 (14.5%)	0.85
*TNFRSF1A*	rs767455	T/T	T/C	C/C	T	C	
		25 (30.1%)	41 (49.4%)	17 (20.5%)	91 (54.8%)	75 (45.2%)	0.80
*TNFRSF1B*	rs1061622	T/T	T/G	G/G	T	G	
		52 (62.7%)	29 (34.9%)	2 (2.4%)	133 (80.1%)	33 (19.9%)	0.67
*TNFRSF1B*	rs1061624	G/G	A/G	A/A	G	A	
		9 (10.8%)	56 (67.5%)	18 (21.7%)	74 (44.6%)	92 (55.4%)	0.16
*TNFRSF1B*	rs3397	T/T	C/T	C/C	T	C	
		33 (39.8%)	37 (44.6%)	13 (15.7%)	103 (62.0)	63 (38.0)	0.92
*TNFRSF1B*	rs976881	C/C	T/C	T/T	C	T	
		38 (45.8%)	30 (36.1%)	15 (18.1%)	106 (63.9)	60 (36.1)	0.61

**Table 3:** SNPs genotypes stratified by treatment effectiveness (SFR vs. non-SFR).

SNP	Genotype	Total	SFR	Non-SFR	*p*-Value
rs3397	WT	29 (40.3%)	14 (32.6%)	15 (51.7%)	0.104
	HOMO	6 (8.3%)	4 (9.3%)	2 (6.9%)	-
	HETE	31 (43.1%)	21 (48.8%)	10 (34.5%)	0.228
rs361525	WT	39 (54.2%)	27 (62.8%)	12 (41.4%)	0.074
	HOMO	12 (16.7%)	5 (11.6%)	7 (24.1%)	0.162
	HETE	19 (26.4%)	10 (23.3%)	9 (31.0%)	0.463
rs1800629	WT	66 (91.7%)	42 (97.7%)	24 (82.8%)	0.025
	HOMO	1 (1.4%)	0 (0.0%)	1 (3.4%)	-
	HETE	5 (6.9%)	1 (2.3%)	4 (13.8%)	-
rs767455	WT	21 (29.2%)	14 (32.6%)	7 (24.1%)	0.441
	HOMO	15 (20.8%)	6 (14.0%)	9 (31.0%)	0.080
	HETE	36 (50.0%)	23 (53.5%)	13 (44.8%)	0.471
rs1061622	WT	44 (61.1%)	27 (62.8%)	17 (58.6%)	0.722
	HOMO	1 (1.4%)	0 (0.0%)	1 (3.4%)	-
	HETE	27 (37.5%)	16 (37.2%)	11 (37.9%)	0.951
rs1799724	WT	56 (77.8%)	31 (72.1%)	25 (86.2%)	0.158
	HOMO	5 (6.9%)	4 (9.3%)	1 (3.4%)	-
	HETE	11 (15.3%)	8 (18.6%)	3 (10.3%)	0.339
rs1061624	WT	8 (11.1%)	3 (7.0%)	5 (17.2%)	0.174
	HOMO	17 (23.6%)	14 (32.6%)	3 (10.3%)	0.029
	HETE	47 (65.3%)	26 (60.5%)	21 (72.4%)	0.296
rs976881	WT	34 (47.2%)	18 (41.9%)	16 (55.2%)	0.267
	HOMO	11 (15.3%)	5 (11.6%)	6 (20.7%)	0.295
	HETE	27 (36.5%)	20 (46.5%)	7 (24.1%)	0.054

**Table 4:** Identification of predictive factors of effectiveness of anti-TNF-α biological drugs.

Characteristics	Univariate OR (95% CI)	*p*-Value	Multivariate OR (95% CI)	*p*-Value
Age	1.00 (0.97–1.23)	0.914	1.00 (0.96–1.04)	0.988
Gender (M)	1.27 (0.47–3.39)	0.639	0.52 (0.14–1.94)	0.326
Diseases				
RCU	ref		ref	
MC	0.50 (0.19–1.33)	0.165	0.57 (0.11–2.84)	0.491
Drugs				
INF	ref		ref	
ADA	0.45 (0.17–1.19)	0.107	0.37 (0.12–1.18)	0.094
Baseline				
HBI/PMS	1.04 (0.93–1.16)	0.490	1.10 (0.96–1.27)	0.178
Steroid therapy	0.54 (0.19–1.54)	0.246	0.86 (0.15–4.80)	0.864
SNPs				
rs3397				
WT	ref		ref	
HOMO	2.14 (0.53–8.72)	0.287	0.43 (0.05–3.68)	0.444
HETE	2.25 (0.79–6.42)	0.129	0.85 (0.16–4.49)	0.843
rs361525				
WT	ref		ref	
HOMO	0.33 (0.09–1.25)	0.102	0.14 (0.03–0.69)	0.016
HETE	0.52 (0.17–1.57)	0.245	0.38 (0.10–1.43)	0.152
rs1800629				
WT	ref		ref	
HOMO	-		-	
HETE	0.14 (0.02–1.35)	0.090	0.09 (0.01–1.00)	0.050
rs767455				
WT	ref		ref	
HOMO	0.33 (0.08–1.32)	0.117	0.10 (0.02–0.60)	0.012
HETE	0.89 (0.29–2.75)	0.832	0.78 (0.21–2.83)	0.699
rs1061622				
WT	ref		ref	
HOMO	-		-	
HETE	0.92 (0.34–2.44)	0.860	0.85 (0.24–3.07)	0.803
rs1799724				
WT	ref		ref	
HOMO	3.23 (0.34–30.72)	0.308	21.96 (0.86–558.87)	0.061
HETE	2.15 (0.52–8.97)	0.293	3.39 (0.63–18.34)	0.16
rs1061624				
WT	ref		ref	
HOMO	7.78 (1.17–51.9)	0.034	5.90 (0.59–58.87)	0.130
HETE	2.06 (0.44–9.65)	0.357	2.02 (0.30–13.74)	0.472
rs976881				
WT	ref		ref	
HOMO	0.74 (0.19–2.90)	0.666	1.30 (0.17–9.88)	0.801
HETE	2.54 (0.85–7.58)	0.095	2.45 (0.45–13.22)	0.298

**Table 5:** SNPs genotypes stratified on the basis of treatment discontinuation (discontinuers vs. completers).

Characteristic	Completers	Discontinuers	*p*-Value
Age (IQR), years	37.5 (28.3–51.5)	38.5 (22.4–53.0)	0.737
Gender (M)	47 (65.3%)	6 (54.5%)	0.490
Diseases			
RU	32 (44.4%)	6 (54.5%)	0.531
CD	40 (55.6%)	5 (45.5%)	
Drugs			
INF	43 (59.7%)	8 (72.7%)	0.409
ADA	29 (40.3%)	3 (27.3%)	
Baseline			
HBI/PMS (IQR)	3 (1–5)	5 (1–7)	0.212
Steroid therapy	49 (68.1%)	7 (63.6%)	0.771
SNPs			
rs3397			
WT	29 (40.3%)	4 (36.4%)	0.739
HOMO	6 (8.3%)	1 (9.1%)	-
HETE	31 (43.1%)	6 (54.5%)	0.475
rs361525			
WT	39 (54.2%)	7 (41.4%)	0.556
HOMO	12 (16.7%)	1 (24.1%)	-
HETE	19 (26.4%)	3 (31.0%)	0.951
rs1800629			
WT	66 (91.7%)	8 (72.7%)	0.060
HOMO	1 (1.4%)	0 (0.0%)	-
HETE	5 (6.9%)	3 (27.3%)	0.033
rs767455			
WT	21 (29.2%)	4 (36.4%)	0.628
HOMO	15 (20.8%)	2 (18.2%)	-
HETE	36 (50.0%)	5 (45.5%)	0.779
rs1061622			
WT	44 (61.1%)	8 (72.7%)	0.458
HOMO	1 (1.4%)	1 (9.1%)	-
HETE	27 (37.5%)	2 (18.2%)	-
rs1799724			
WT	56 (77.8%)	8 (72.7%)	0.710
HOMO	5 (6.9%)	0 (0.0%)	-
HETE	11 (15.3%)	3 (27.3%)	0.322
rs1061624			
WT	8 (11.1%)	1 (9.1%)	-
HOMO	17 (23.6%)	1 (9.1%)	-
HETE	47 (65.3%)	9 (81.8%)	0.275
rs976881			
WT	34 (47.2%)	4 (36.4%)	0.501
HOMO	11 (15.3%)	4 (36.4%)	0.091
HETE	27 (37.5%)	3 (27.3%)	0.511

**Table 6:** Summary of key findings.

Gene	rs Number	Genotype	Clinical Implications	*p*-Value
*TNF-α*	rs1800629	G/A and A/A	Increased risk of discontinuing biologics	0.047
*TNF-α*	rs361525	A/A	Reduced probability of achieving SFR	0.016
*TNFRSF1A*	rs767455	C/C	Reduced probability of achieving SFR	0.012

## Data Availability

The raw data supporting the conclusions of this article will be made available by the authors upon request.
